# HLA B subtype analysis in patients developing tuberculosis during anti-TNF treatment

**DOI:** 10.1038/s41598-025-00290-1

**Published:** 2025-04-30

**Authors:** Fatih Albayrak, Gökhan Keser, Serdar Oztuzcu, Belkıs Nihan Coşkun, Yavuz Pehlivan, Tuba Demirci Yıldırım, Gökçe Kenar Artın, Gözde Çetin Yıldırım, Bünyamin Kısacık

**Affiliations:** 1 Department of Rheumatology, Gaziantep University Hospital, Osmangazi Neighborhood, Şehitkamil, Gaziantep, Turkey; 2 Department of Rheumatology, Ege University Faculty of Medicine Hospital, İzmir, Turkey; 3 Department of Rheumatology, Uludağ University Faculty of Medicine Hospital, Bursa, Turkey; 4 Department of Rheumatology, Dokuz Eylul University Faculty of Medicine Hospital, İzmir, Turkey; 5 Department of Rheumatology, Kahramanmaraş University Faculty of Medicine Hospital, Kahramanmaraş, Turkey; 6 Department of Rheumatology, Sanko University Medical Faculty Hospital, Gaziantep, Turkey

**Keywords:** HLA-B genes, Tumor necrosis factor inhibitors, Tuberculosis, Molecular medicine, Rheumatology

## Abstract

In this study, we aimed to evaluate HLA-B subtypes in cases developing TB while using anti-TNF agents and to compare with the HLA-B subtypes of three different groups including community-acquired TB cases, anti-TNF patients without TB and healthy controls. The study consisted of four groups. Group 1: 35 community-acquired TB patients, group 2; 47 anti-TNF-TB patients, group 3; 61 anti-TNF (non TB) patients and group 4; 40 healthy controls. HLA-B subgroups were studied genetically from blood samples taken from the patients. Approval was received from the ethics committee. HLA-B typing was performed by using the LIFECODES HLA-B eRES SSO Typing SSO typing kit (Immucor, Stamford, CT, USA). Among HLA-B subtypes, significant differences between the four groups were found only for HLA-B5, HLA-B8, HLA-B27 and HLA-B38. Although frequency of HLA-B8 was highest in patients developing TB during anti-TNF treatment, significance was lost after Bonferoni correction. HLA-B27 was significantly higher in both anti-TNF groups, with and without TB, due to presence of patients with ankylosing spondylitis in those groups. To our knowledge, this is the first study analyzing HLA-B subtypes in anti-TNF patients developing TB. Although in literature HLA-B8 positivity was suggested to associate with or to confer a risk for community-acquired TB, we could not confirm this association in anti-TNF patients developing TB. However, further studies with larger number of patients from different ethnic groups are clearly needed to get the final word in this topic.

## Introduction

Anti-tumor necrosis factor (anti-TNF) therapies have long been used in the treatment of rheumatologic diseases^1^. In particular, they are recommended in the treatment guidelines for rheumatoid arthritis (RA), ankylosing spondylitis (AS) and psoriatic arthritis (PsA) in cases resistant to first-line therapies. Although anti-TNF drugs are widely used in different inflammatory diseases, there are some limitations to their use such as opportunistic infections^2^. Especially reactivation of latent tuberculosis (TB) can still pose a significant problem when using these therapeutic agents. Despite precautions to prevent TB, new cases of TB are still reported. In a previous multicenter study, we showed that isoniazid prophylaxis for less than nine months and the use of infliximab among the five different anti-TNF agents were independent risk factors for the development of TB^3^.

Human leukocyte antigens (HLA)-B-related genetic mutations have been shown to be associated with the development of community-acquired TB. Associations between HLA-B8, -B18, -B5, and -B27 positivity with the development of community-acquired TB have been reported. In addition, the familial cases reported support that genetic structure may be a risk factor for the development of TB^4^. Another interesting finding that attracted our attention was higher frequency of TB occurrence among Behçet’s patients receiving anti-TNF agents^3^. This observation might implicate possible role of HLA-B subtypes for the development of TB during anti-TNF treatment. Currently, the guidelines do not have any TB-related recommendations other than screening for latent TB before starting anti-TNF treatment. However, genetic predisposition for TB development is obviously important, especially in patients in the risk group. Hereby, we aimed to evaluate HLA-B subtypes in cases developing TB under anti-TNF treatments and to compare with the HLA-B subtypes of three different groups including community-acquired TB cases, anti-TNF patients without TB and healthy controls.

## Material-methods

### Patient selection

The study was designed with four groups. Group 1: community-acquired tuberculosis, Group 2: those who developed tuberculosis while under Anti-TNF treatment, Group 3: those who did not develop tuberculosis while under Anti-TNF treatment, and Group 4: healthy controls.

We planned the study design accordingly. We used the following methods to diagnose active tuberculosis;

- Mycobacterium tuberculosis bacilli were demonstrated in sputum and/or bronchoalveolar lavage samples by Erlich Ziehl Neelsen (EZN) acid-fast bacteria staining method.
- Mycobacterium tuberculosis is seen in the bacteriological culture of a sample taken from an organ and/or tissue where tuberculosis is seen.
- The presence of Mycobacterium tuberculosis was demonstrated by demonstrating polymerase chain reaction (PCR) in the sample and histopathological morphology compatible with tuberculosis in the biopsy material.

Group 1 included 35 consecutive patients who were diagnosed and treated for community-acquired TB at a tuberculosis dispensary. Community-acquired TB cases consist of patients of similar age and gender who were found to have Mycobacterium tuberculosis culture positivity, AFB positivity or Mycobacterium tuberculosis PCR positivity. Demographic and clinical characteristics of the patients were recorded by accessing their files.

Group 2 included 47 patients who developed TB while receiving anti-TNF treatment. All of them were being followed up in different rheumatology departments in Turkey. Diagnosis of TB was made based upon Mycobacterium tuberculosis culture positivity, ARB positivity or Mycobacterium tuberculosis PCR positivity, positive quantiferon gama test and clinical as well as radiological evidence of TB. In addition to appropriate clinical presentation, TB culture positivity, ARB positivity, or Mycobacterium tuberculosis PCR positivity was taken as the main criterion. Cases that did not clearly match the clinical picture or had only QuantiFERON or PPD positivity were not included in the study.

Group 3 included 61 patients receiving anti-TNF treatment and did not develop TB during follow-up period for at least 48 months. At the time of entry, quantiferon gama test was negative in all of them. For patients in this group, Anti-TNF use for less than 24 months was considered as an exclusion criterion.

Healthy controls (Group 4) included subjects with no disease history. A control group was taken from each center at a ratio of approximately 1:1 for all cases of tuberculosis developing under TNF in the centers participating in the study. PPD test was performed and 40 subjects with a PPD result < 5 mm were accepted as members of the healthy control group and included in the study. The healthy control group consisted of medical students, nurses and hospital employees.

Although this study is actually a prospective study, the patients’ past demographic characteristics, disease duration, treatments, related laboratory and imaging data were collected retrospectively. All cases from the date anti-TNF drugs were introduced to our country until December 2023 were included in the study.All of the patients in three groups and healthy controls were informed about the study and their consent was obtained in accordance with ethical rules. All of them gave consent to participate in the study and three cc of blood was collected from all study participants from the venous vein and placed in a purple hemogram tube and stored at − 80 degrees Celsius in the same way.

### DNA extraction and HLA genotyping

Genomic DNA was extracted from the peripheral blood samples by using the QuickGene-Mini80 DNA Isolation System (Fujifilm, Tokyo, Japan). DNA concentration and purity were determined by Nanodrop spectrophotometer (ThermoScientific, USA) and preserved at −70°C. HLA-B typing was performed by using the LIFECODES HLA-B eRES SSO Typing SSO typing kit (Immucor, Stamford, CT, USA). PCR mixture was prepared with 15 µL of the LIFECODES Master Mix (Immucor), 200 ng of genomic DNA, and 2.5 U Taq polymerase in a final volume of 50 µL and then treated with the following: denaturation at 95°C for 5 min; 40 cycles of amplification (8 cycles: 95°C for 30 s, 60°C for 45 s, 72°C for 45 s, and 32 cycles: 95°C for 30 s, 63°C for 45 s, 72°C for 45 s); and extension at 72°C for 15 min. Hybridization was performed under the following conditions: 97°C for 5 min, 47°C for 30 min, and 56°C for 10 min with 15 µL probe mix and 5 µL of the PCR product. The samples were diluted with 170 µL of the 1:200 pre-diluted streptavidin-phycoerythrin solution and detected within 30 min by using the Luminex 200 system (Luminex Corp.) and were analyzed by MatchIT DNA software (Immucor GTI diagnostics inc. USA) according to the instructions mentioned in the software manual. HLA-B subgroup of the groups analyzed in the present study included HLA-B5, HLA-B7, HLA-B8, HLA-B13, HLA-B14, HLA-B15, HLA-B18, HLA-B27, HLA-B35, HLA-B37, HLA-B38, HLA-B39, HLA-B40, HLA-B41, HLA-B42, HLA-B44, HLA-B45, HLA-B48, HLA-B49, HLA-B50, HLA-B51, HLA-B52, HLA-B53, HLA-B55, and HLA-B57.

### Statistical analysis

Statistical analysis was performed using the IBM-SPSS version 26 software (Armonk, New York, USA). Data were presented as percentages and mean ± standard deviation. For the comparison of categorical data, the chi-square test with the Likelihood Ratio method was used. Since there were four groups, the p-value was adjusted using the Bonferroni correction, with the significance threshold set at *P* < 0.0125. P-values equal to or greater than this threshold were considered non-significant, and pairwise comparisons were performed if overall significance was found.For continuous data across the four groups, a one-way ANOVA was conducted. The assumptions of normality and homogeneity of variances were checked using the Shapiro-Wilk test and Levene’s test.

For the comparison of four groups, the p-value was adjusted using the Bonferroni correction, and the significance threshold was set at < 0.0125. P-values equal to or greater than this threshold were considered as non-significant. In the event of significance in the overall comparisons, we proceeded with pairwise group comparisons to identify which specific groups contributed to the observed significance. For the comparison of the four groups, we utilized the chi-square test. In assessing the significance of the p-value, we opted for the Likelihood Ratio method as it is well-suited for testing the association between categorical variables, considering the characteristics of our data.

### Ethics

The responsible unit of TB dispensary under the provincial health directorate was informed about the present study and permission was obtained from the relevant unit. In addition, ethics committee approval was obtained from Gaziantep University Ethics Committee. The ethical approval was obtained from Gaziantep University Ethics Committee Board (https://doi.org/10-2011/182, 25/10/2011). All methods were performed in accordance with the relevant guidelines and regulations by including a statement in the methods.

## Results

The study consisted of four groups. Group 1: 35 community-acquired TB patients, group 2; 47 anti-TNF-TB patients, group 3; 61 anti-TNF (non TB) patients and group 4; 40 healthy controls. Of the study participants, 71 (38.8%) were female and 112 (61.2%) were male. Detailed demographic characteristics of the study groups were shown in Table 1.

**Table 1:** Clinical and demographic characteristics of the groups included in the study

	Group 1 (Community TB)	Group 2 (Anti-TNF-TB)	Group 3 (Anti-TNF-nonTB)	Group 4 (Healthy Control)	*p*
N	35	47	61	40	-
Gender (male/female)	18/17	26/21	43/18	25/15	NS
Mean age (years ± SD)	43.3 ± 9.2	42.9 ± 12.6	41.0 ± 10.4	38.7 ± 6.4	0.158

TB: Tubercolosis

In patients who used used anti-TNF agents and developed TB, disease distribution was as follows: ankylosing spondylitis 23 (48.9%), rheumatoid arthritis 13 (27.7%), Behçet’s syndrome 8 (17%) and psoriatic arthritis 3 (6.4%). Anti-TNF drugs used in those cases included adalimumab 20 (42.6%), infliximab 15 (31.9%), etanercept 7 (14.9%) and certolizumab pegol 5 (10.6%). Anti-TNF agents used in patients who did not develop TB were adalimumab, infliximab (IFX), etanercept (ETN) and certolizumab pegol in 29 (47.5%), 21 (34.4%), 8 (13.1%) and 3 (4.9%) cases, respectively, with no statistical significance. Among patients developing TB while using anti-TNF agents, 46.8% had pulmonary involvement and 53.2% had extra-pulmonary involvement including lymphadenitis TB, military TB, peritoneal TB, intestinal TB and bone TB). In community-acquired TB cases, pulmonary and extra-pulmonary involvements were 65% and 35% respectively. Frequency of extra-pulmonary involvement was significantly higher in anti-TNF group compared with community acquired group. There was no significant difference between anti-TNF patients with and without TB with respect to age, INH prophylaxis, INH regular use, tuberculin skin test (TST) positivity and Quantiferon test positivity. The only significant difference was the duration of anti-TNF use (p:0.001) (Table 2).

**Table 2:** Clinical features of Anti-TNF patients with and without TB)

	Anti-TNF Patients with TB	Anti-TNF Patients without TB	*P*
N	47	61	–
Gender (male/female)	26/21	43/18	NS
Mean age (years ± SD)	42.9 ± 12.6	41.0 ± 12.6	NS
Duration of anti-TNF usage (months)	22.3 ± 16.2	43.8 ± 4.6	0.001
TST positivity, n (%)	34 (72.3)	49 (80.3)	0.329
Quantiferon positivity n(%)	4 (8.5)	3 (3.9)	0.744
Isoniazid prophylaxis, n (%)	38 (78.7)	52 (85.2)	0.377
Patients used INH regularly, n (%)	32 (84.2)	49 (94.2)	0.118
Diseases			
Rheumatoid arthritis, n (%)	13 (27.7)	6 (9.8)	
Ankylosing spondylitis, n (%)	23 (48.9)	51 (83.6)	
Psoriatic arthritis, n (%)	3 (6.4)	–	
Behçet’s syndrome, n (%)	8 (17.0)	4 (6.6)	
Anti-TNF			0.699
Adalimumab	20 (42.6)	29 (47.5)	
Infliximab	15 (31.9)	21 (34.4)	
Etanercept	7 (14.9)	8 (13.1)	
Certolizumab pegol	5 (10.6)	3 (4.9)	

TB: Tuberculosis, INH: Isoniaside, TST: Tuberculosis skin test, Anti-TNF: Tumor necrosis factor alpha inhibitor.

The initial evaluation of HLA-B subtypes between the four groups showed significant differences between the groups only for HLA-B5, HLA-B8, HLA-B27 and HLA-B38, as shown in Table 3. Especially, HLA-B8 positivity was significantly higher among Group 2 (Anti-TNF-TB group), i.e. those developing TB under anti-TNF treatment, compared to other study groups (p: 0.040). However, after correction with Bonferoni, only HLA-B27 frequency exhibited significance. Pairwise comparisons revealed significantly higher prevalence of total TB in the Anti-TNF-TB group compared to healthy subjects and patients treated with anti-TNF without TB. Regarding HLA-B27, both anti-TNF-TB group and anti-TNF group without TB demonstrated significantly higher rates compared to two other groups, but no significant difference was observed between the anti-TNF patients with and without TB. Given that 48.9% of the patients in anti-TNF-TB group and 83.6% of the patients in anti-TNF group without TB had AS, increased frequency of HLA-B27 positivity was an expected finding in both of those groups.

**Table 3:** Frequencies of HLA-B subgroups in study groups

HLA Antigens	Anti-TNF (TB) *N*	Community TB *N*	Anti-TNF (non-TB) *N*	Control (Healthy) *N*	*P*
B5	**11**	**21**	**25**	**15**	**0.018**
B7	7	2	6	7	0.368
B8	**8**	**1**	3	2	**0.040**
B13	3	2	3	2	0.985
B14	1	0	3	**4**	0.159
B15	2	**2**	2	3	0.803
B18	4	4	7	3	0.898
B27	**14**	2	**30**	4	**0.001**
B35	12	9	12	13	0.544
B37	0	0	0	1	0.312
B38	**3**	**6**	**2**	**1**	**0.037**
B39	1	0	1	1	0.839
B40	2	5	5	3	0.450
B41	1	3	1	1	0.279
B42	0	1	0	0	0.238
B44	5	6	9	5	0.857
B45	0	0	0	1	0.312
B48	0	0	1	0	0.574
B49	1	5	5	5	0.208
B50	1	2	3	2	0.859
B51	14	10	14	10	0.829
B52	2	5	3	2	0.242
B53	0	0	1	0	0.574
B55	0	4	5	2	0.150
B57	1	1	1	1	0.981

Bold indicates statistical significance.

## Discussion

To our knowledge, this is the first study in the literature evaluating HLA-B subtypes in cases developing TB while using anti-TNF agents and comparing with the HLA-B subtypes of three different groups including community-acquired TB cases, anti-TNF patients without TB and healthy controls. We found that HLA B8 positivity was highest in patients developing TB while receiving anti-TNF agents. However, significance disappeared after correction analysis with Bonferoni. As expected, HLA-B27 was significantly higher in both anti-TNF groups, with and without TB, due to presence of patients with ankylosing spondylitis in those groups. In consistent with the previous literature, we also found that TB caused by anti-TNF was more frequently extra-pulmonary, while community-acquired TB preferentially involved the lungs.

The first alert regarding the increased TB risk with anti-TNF therapy was issued by the Adverse Event Reporting System of the FDA. TB was seen at a rate of 144/100,000 patients receiving IFX per year and 35/100,000 patients receiving ETN per year. TB frequency was 5.2–6.8 for 100,000 cases per year in the general US population (5.). In Turkey, this risk may be especially pronounced, since the background TB incidence is about 25–49 new cases in every 100,000 people^6^.

The mechanism involved in the development of latent TB under anti-TNF therapy is not well understood. Mycobacterial antigens expressed on the surface of antigen-presenting cells are presented to T and B lymphocytes in the context of HLA class I and II antigens to induce specific immunity in TB^7^. In this respect, genetically determined different HLA-B subtypes may constitute a risk for TB development. Besides, Papiha et al. previously reported the relationship between phosphoglucomutase enzyme and TB development risk^8^. Phosphoglucomutase enzyme synthesis is controlled by a gene located in the major histocompatibility complex (MHC) region on the short arm of the sixth chromosome. Based on this finding, many studies were performed to find out a relationship between TB and HLA-A, -B and -C alleles in different ethnic populations. In brief, possible links with HLA-B8, -B15, -B18, -B27 and -B35 genes located at the B locus were reported for community-acquired TB^9–11^.

Selby et al. found that the frequency of HLA-B8 was 56% in patients with TB, being significantly higher compared to healthy controls (*p* < 0.001)^12^. In another study, the frequency of HLA-B8 was found to be 19.5% in TB cases (12.). These studies performed in general population may suggest that HLA-B8 positivity may confer a risk or a predisposition to community-acquired TB. However, in the present study, we could not confirm HLA-B8 association in anti-TNF patients developing TB.

HLA-B5 positivity is known to be high in healthy people in countries on the Silk Road^14^. The HLA-B5 positivity rate in healthy individuals in Turkey has been determined to be 10%^14^.Its association with community-acquired TB has been previously shown. Hwang et al. found that the frequency of HLA-B5 was significantly higher in Black Americans with TB^15^. On the other hand, HLA-B5 positivity is common in Behçet’s syndrome and Kısacık et al. found a higher prevalence of TB in patients with Behçet’s disease and HLA-B5 positivity^3^. We also found highest frequency of HLA-B5 in community-acquired TB cases in the present study. However, frequency of HLA-B5 in patients developing TB under anti-TNF treatment (Group 2) was lower than expected in the present study.

HLA-B38 was first identified in 2006 (15.). It has been associated with the pathogenesis of some autoimmune diseases. Preliminary studies have linked HLA-B38 to protection from multiple sclerosis and pemphigus vulgaris^17^. In literature, there are no studies on HLA-B38 and TB risk. This study we have conducted is the first in this regard. We found that HLA B38 was highest in community-acquired TB group, followed by anti-TNF TB group, however low number of total patients with HLA-B38 positivity and lack of significance do not let as draw a conclusion. In a study conducted in our country with 142 blood donors, HLA-B*35, B*51 and B*44 were the most frequent ones among the HLA-B alleles^18^. In our study, HLA-B*5, HLA-B*51, HLA-B*27, HLA-B*35 and HLA-B*44 were the most frequently found alleles. It was found to be similar to the other study.

Our study has some limitations. Firstly; whole genome analysis could have been more effective to evaluate the relationship more in detail, rather than single HLA-B subtyping. However, we could not perform whole genome analysis, because of its high cost. The second limitation is the need for larger patient numbers for better results in genetic studies. However, the number of cases developing TB has decreased considerably due to more stringent TB screening and increased awareness of TB risk. It seems very difficult to reach high number of patients developing TB under anti-TNF treatment. Third limitation is that HLA subtypes differ in patients from different geographical and ethnic backgrounds.

In conclusion; to our knowledge, this is the first study in which HLA-B subtype alleles were analyzed in patients developing TB under anti-TNF treatment. Although in literature HLA-B8 positivity was suggested to associate with or to confer a risk for community-acquired TB, we could not confirm this association in anti-TNF patients developing TB. However, further studies with larger number of patients from different ethnic groups are clearly needed to get the final word for this topic.

## Data Availability

Data are available upon a reasonable request. The corresponding author can be contacted when data related to this study is requested.
